# Development of an anti-CDH15/M-cadherin monoclonal antibody Ca_15_Mab-1 for flow cytometry, immunoblotting, and immunohistochemistry

**DOI:** 10.1016/j.bbrep.2025.102138

**Published:** 2025-07-11

**Authors:** Rena Ubukata, Hiroyuki Suzuki, Tomohiro Tanaka, Mika K. Kaneko, Yukinari Kato

**Affiliations:** Department of Antibody Drug Development, Tohoku University Graduate School of Medicine, 2-1 Seiryo-machi, Aoba-ku, Sendai, Miyagi, 980-8575, Japan

**Keywords:** CDH15, M-cadherin, Monoclonal antibody, Cell-Based Immunization and Screening, Flow cytometry, Immunohistochemistry

## Abstract

Cadherins are key cell adhesion molecules that engage in extracellular homophilic binding. CDH15/M‐cadherin is localized to the apical surface of muscle satellite cells (SCs), which play a critical role in tissue regeneration after injury. Although CDH15 is considered a marker of SCs, there is no anti-CDH15 monoclonal antibody (mAb) suitable for flow cytometry. We developed anti-CDH15 mAbs using the Cell-Based Immunization and Screening (CBIS) method containing a flow cytometry-based high-throughput screening. In flow cytometry, a clone Ca_15_Mab-1 (IgG_1_, κ) reacted with human CDH15-overexpressed Chinese hamster ovary-K1 (CHO/CDH15) cells. Furthermore, Ca_15_Mab-1 recognizes endogenous CDH15-expressing human osteosarcoma (Saos-2) and mouse myoblast (C2C12) cell lines. The dissociation constant values of Ca_15_Mab-1 for CHO/CDH15, Saos-2, and C2C12 were determined as 6.1 × 10^−10^ M, 7.9 × 10^−10^ M, and 9.8 × 10^−10^ M, respectively. Furthermore, Ca_15_Mab-1 can detect endogenous CDH15 in immunoblotting and immunohistochemistry. Ca_15_Mab-1, established by the CBIS method, is versatile for basic research and is expected to contribute to clinical studies such as antibody therapy.

## Highlights

• A novel anti-CDH15/M-cadherin monoclonal antibody, Ca_15_Mab-1 was developed by the Cell-Based Immunization and Screening method.
• Ca_15_Mab-1 is useful for the detection of human and mouse CDH15 with high affinity by flow cytometry.
• Ca_15_Mab-1 is also available for immunoblotting and immunohistochemistry.
• Ca_15_Mab-1 would contribute to the basic research for the identification and isolation of muscle satellite cell.
• Ca_15_Mab-1 could be used in the preclinical study of muscular diseases.

## 1 Introduction

Cadherins are a key class of cell adhesion molecules that engage in extracellular homophilic binding [1]. The epithelial cell-cell adhesion molecule cadherin 1 (CDH1/E‐cadherin) forms the adherens junctions and plays a critical role in tumor development [2,3]. CDH1 is a classical cadherin with five extracellular cadherin repeats, a single transmembrane domain, and a cytoplasmic domain. Currently, the cadherin family has more than 100 members in humans, including classical cadherins, protocadherins, and cadherin‐related proteins [4]. Phylogenetic analysis showed that classical cadherins include several distinct types. CDH1, CDH2/N (neuronal)‐cadherin, CDH3/P (placental)‐cadherin, CDH4/R (retinal)‐cadherin, and CDH15/M (muscle)‐cadherin are classified into the type I cadherin [5]. The intracellular domains of cadherins directly interact with β- and γ-catenin (also known as plakoglobin), which subsequently associate with α-catenin. α-Catenin connects to the F-actin cytoskeleton, contributing to cortical tension and the dynamic stability of cell-cell junctions [6,7].

CDH2 and CDH15 are expressed during skeletal muscle development [8]. CDH2 is present throughout muscle lineage development from uncommitted progenitor cells to proliferative myoblasts and nascent myofibers [9]. Although its expression decreases in adult myofibers, it is strongly re-expressed during muscle regeneration, following a pattern similar to that seen in development [9]. CDH15 was initially identified in mouse myoblast C2C12 [10], and the expression starts at the myoblast stage and is primarily restricted to the skeletal muscle lineage [11]. In adult muscle, CDH15 is localized to the apical satellite cell (SC) membrane, which is in direct contact with the myofiber plasma membrane [12,13].

Skeletal muscle SCs are responsible for the regeneration of skeletal muscle [14,15]. SCs typically remain in a long-term quiescent state and express PAX7, a transcription factor essential for maintaining this state [16]. Upon muscle injury, SCs become activated and initiate the expression of myogenic transcription factors MYOD and MYF5, which promotes proliferation as transit-amplifying myoblasts [[17], [18], [19]]. These myoblasts then differentiate and fuse with each other and with pre-existing myofibers to facilitate tissue repair [[17], [18], [19]]. A portion of SCs undergoes self-renewal, allowing muscles to regenerate through multiple cycles of SC-dependent repair [[17], [18], [19]]. However, when SC quiescence is abnormally disrupted, such as in aging, it often results in the depletion of the functional SC pool and compromised regenerative capacity [[20], [21], [22], [23]].

Both CDH2 and CDH15 are believed to be involved in lineage determination, myoblast differentiation, and myoblast fusion [8]. However, CDH15-knockout (KO) mice displayed no noticeable defects in muscle development or regeneration [24]. CDH2-KO mice did not survive beyond embryonic day 10. However, cultured somites from the CDH2-KO embryos still formed elongated cells that expressed muscle markers and maintained intact adherens junctions [25]. These results suggest that neither CDH2 nor CDH15 is strictly required for myogenesis, and they may serve redundant roles in muscle development and regeneration. Therefore, the conditional depletion of CDH2 and CDH15 in SCs was performed. The CDH2 and CDH15 double KO SCs exhibited a state resembling an early stage of SC activation, suggesting that CDH2 and CDH15-mediated adhesion to neighboring cells mediates the SC quiescence [26]. Since the CDH15 expression is restricted in SC, a monoclonal antibody (mAb) against CDH15 is thought to help identify or isolate SC cells from muscle. Although several anti-CDH15 mAbs have been developed for immunoblotting [27] and immunofluorescence [12], there is no anti-CDH15 mAb suitable for flow cytometry.

We have developed various mAbs against membrane proteins such as receptor tyrosine kinases [[28], [29], [30]] and chemokine receptors [[31], [32], [33]] using the Cell-Based Immunization and Screening (CBIS) method. The CBIS method includes immunizing antigen-overexpressed cells and flow cytometry-based high-throughput screening. Therefore, mAbs obtained by the CBIS method tend to recognize conformational epitopes and are suitable for flow cytometry. Furthermore, some of these mAbs also apply to immunoblotting and immunohistochemistry (IHC). This study employed the CBIS method to generate highly versatile anti-CDH15 mAbs.

## 2 Materials and methods

### 2.1 Cell lines

Chinese hamster ovary (CHO)–K1, mouse myeloma P3X63Ag8U.1 (P3U1), human glioblastoma LN229, human osteosarcoma Saos-2, and mouse myoblast C2C12 were obtained from American Type Culture Collection (ATCC, Manassas, VA, USA). LN229, Saos-2, and C2C12 were maintained in Dulbecco's Modified Eagle Medium (Nacalai Tesque, Inc., Kyoto, Japan), supplied with 100 U/mL penicillin, 100 μg/mL streptomycin, 0.25 μg/mL amphotericin B (Nacalai Tesque, Inc.), and 10% heat-inactivated fetal bovine serum (FBS; Thermo Fisher Scientific, Inc., Waltham, MA, USA). CHO–K1 and P3U1 were maintained in Roswell Park Memorial Institute-1640 medium (Nacalai Tesque, Inc.), supplied with 100 U/mL penicillin, 100 μg/mL streptomycin, 0.25 μg/mL amphotericin B, and 10% heat-inactivated FBS. All the cells were cultured in a humidified incubator at 37 °C with 5% CO_2_.

### 2.2 Plasmid construction and establishment of stable transfectants

The genes encoding human *CDH1* (NM_004360) and *CDH3* (NM_001793.6) cloned into pCMV6neo-myc-DDK vector were purchased from OriGene Technologies, Inc. (Rockville, MD). The genes encoding human *CDH2* (NM_001792) and *CDH15* (NM_004933) were obtained from the RIKEN BioResource Research Center (Ibaraki, Japan). The gene encoding human *CDH4* (NM_001794.5) was synthesized by Eurofins Genomics KK (Tokyo, Japan). The propeptide-deleted *CDH2*, *CDH4*, and *CDH15* cDNAs with an N-terminal PA16 tag (GLEGGVAMPGAEDDVV) [34] were subcloned into the pCAG-Ble vector (FUJIFILM Wako Pure Chemical Corporation, Osaka, Japan). The propeptide-deleted *CDH15* cDNA with an N-terminal MAP16 tag (PGTGDGMVPPGIEDKI) [35] was subcloned into the pCAG-Ble vector. Using the Neon transfection system, these plasmids were transfected into LN229 or CHO–K1 cells (Thermo Fisher Scientific, Inc.). The transfectants were sorted using an anti-CDH1 mAb (clone DECMA-1, BioLegend, San Diego, CA, USA), an anti-CDH3 mAb (clone MM0508-9V11, Abcam, Cambridge, UK), an anti-PA16 tag mAb (clone NZ-1) [34], and an anti-MAP16 tag mAb (PMab-1) [35] using a SH800 cell sorter (Sony Corporation, Tokyo, Japan). The transfectants were maintained in a medium containing 0.5 mg/mL of Zeocin (InvivoGen, San Diego, CA, USA) or 0.5 mg/mL of G418 (Nacalai Tesque, Inc.). Finally, we established the CDH-overexpressed stable transfectants: CHO/CDH1, CHO/PA16-CDH2 (CHO/CDH2), CHO/CDH3, CHO/PA16-CDH4 (CHO/CDH4), CHO/PA16-CDH15 (CHO/CDH15), and LN229/MAP16-CDH15.

### 2.3 Hybridoma production

The female BALB/cAJcl mice were purchased from CLEA Japan (Tokyo, Japan). Animal experiments were approved by the Animal Care and Use Committee of Tohoku University (Permit number: 2022MdA-001). They were carried out following the NIH (National Research Council) Guide for the Care and Use of Laboratory Animals. The mice were intraperitoneally immunized with LN229/MAP16-CDH15 cells (1 × 10^8^ cells/injection) and Alhydrogel adjuvant 2 % (InvivoGen). After three additional immunizations per week (1.5–2.0 × 10^8^ cells/injection), a booster injection (2.0 × 10^8^ cells/injection) was administered two days before harvesting the spleen cells from immunized mice. The hybridomas were generated as described previously [31]. The supernatants, which were positive for CHO/CDH15 and negative for CHO–K1, were screened by an SA3800 Cell Analyzer (Sony Corporation, Tokyo, Japan).

### 2.4 Flow cytometry

Cells were harvested using 1 mM ethylenediaminetetraacetic acid (EDTA; Nacalai Tesque, Inc.). The cells (1 × 10^5^ cells) were washed with 0.1 % bovine serum albumin (BSA) in phosphate-buffered saline (PBS, blocking buffer) and treated with mAbs for 30 min at 4 °C. The cells were then stained with anti-mouse IgG or anti-rat IgG conjugated with Alexa Fluor 488 (2000-fold dilution; Cell Signaling Technology, Inc., Danvers, MA, USA) for 30 min at 4 °C. The data (5000 events) were collected using an SA3800 Cell Analyzer. Cells were gated on the dot plot based on side scatter (SSC) and forward scatter (FSC), and the fluorescence intensity was determined analyzed using FlowJo software (BD Biosciences, Franklin Lakes, NJ, USA).

### 2.5 Determination of dissociation constant values using flow cytometry

CHO/CDH15, Saos-2, and C2C12 (2 × 10^5^ cells) were treated with serially diluted Ca_15_Mab-1. Subsequently, the cells were treated with anti-mouse IgG-conjugated with Alexa Fluor 488 (200-fold dilution) for 30 min at 4 °C. The data (10,000 events) were collected using BD FACSLylic (BD Biosciences) and the geometric mean (GeoMean) was determined using FlowJo. The fitting binding isotherms (vertical axis, GeoMean; horizontal axis, mAb concentration) determined the dissociation constant (*K*_D_) values to built-in one-side binding models of GraphPad Prism 6 (GraphPad Software, Inc. La Jolla, CA, USA).

### 2.6 Immunoblotting

Whole-cell lysates (10 μg of protein) were separated into polyacrylamide gels and transferred onto polyvinylidene difluoride membranes (Merck KGaA, Darmstadt, Germany). The membranes were blocked with 4% skim milk (Nacalai Tesque, Inc.) in PBS containing 0.05% Tween 20 and incubated with 1 or 10 μg/mL of Ca_15_Mab-1, 1 μg/mL of NZ-1, or 1 μg/mL of an anti-β-actin mAb (clone AC-15; Sigma-Aldrich Corporation, St. Louis, MO, USA). Then, the membranes were incubated with anti-mouse IgG or anti-rat IgG conjugated with horseradish peroxidase (1000-fold dilution; Agilent Technologies, Inc., Santa Clara, CA, USA). Chemiluminescence signals were developed and detected as described previously [29].

### 2.7 Immunohistochemistry (IHC) using cell blocks

Cells were fixed with 4% paraformaldehyde, and the cell blocks were prepared using iPGell (Genostaff Co., Ltd., Tokyo, Japan) (FUJIFILM Wako Pure Chemical Corporation). The formalin-fixed paraffin-embedded (FFPE) cell sections were stained with Ca_15_Mab-1 (0.1 or 1 μg/mL) or NZ-9, which was produced using the V_H_ of NZ-1, C_H1_, C_H2_ and C_H3_ of mouse IgG_2a_, and the light chain of NZ-1, (1 μg/mL) using BenchMark ULTRA PLUS with the ultraView Universal DAB Detection Kit (Roche Diagnostics, Indianapolis, IN, USA).

## 3 Results

### 3.1 Development of anti-CDH15 mAbs

**Fig. 1:**
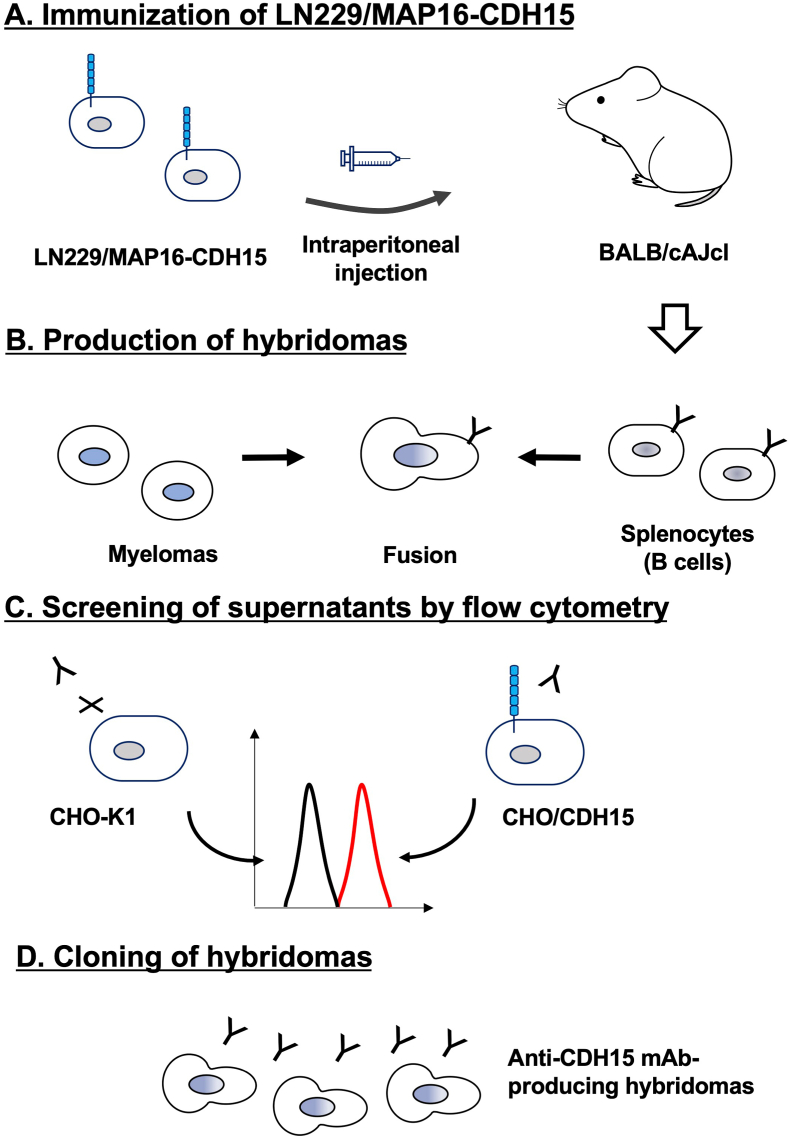
Schematic representation of anti-CDH15 mAbs production. (A) LN229/MAP16-CDH15 was injected intraperitoneally into BALB/cAJcl mice. (B) After five immunizations per week, spleen cells were fused with P3U1. (C) The supernatants from hybridomas were screened by flow cytometry using CHO/CDH15 and CHO–K1 cells. (D) Anti-CDH15-specific mAb-producing hybridoma clones (Ca_15_Mabs) were established through limiting dilution.

Two BALB/cAJcl mice were immunized with LN229/MAP16-CDH15 cells, and the generated hybridomas were seeded into 96-well plates. After forming colonies, supernatants were subject to flow cytometry-based high throughput screening to identify supernatants that were positive for CHO/CDH15 and negative for CHO–K1. The screening resulted in 29 positive wells out of 956 wells (3.0%). Subsequently, anti-CDH15 mAb-producing hybridomas were cloned by limiting dilution. Finally, clones Ca_15_Mab-1 (IgG_1_, κ), Ca_15_Mab-2 (IgG_1_, κ), Ca_15_Mab-3 (IgG_1_, κ), Ca_15_Mab-5 (IgG_1_, κ), and Ca_15_Mab-8 (IgG_1_, κ) were established (Fig. 1).

### 3.2 Flow cytometry using Ca_15_Mabs

**Fig. 2:**
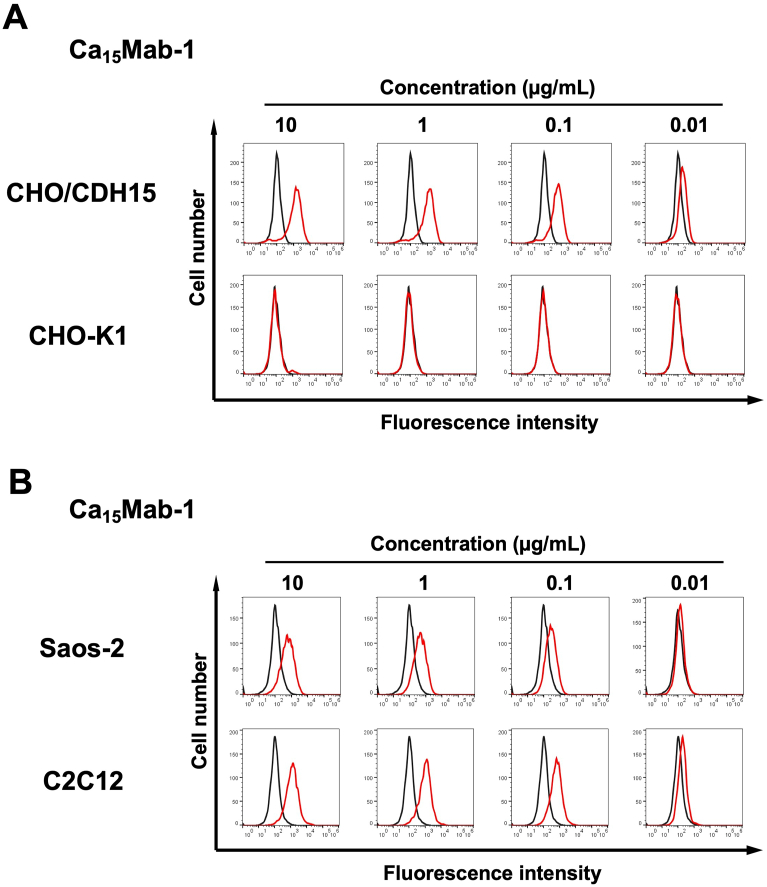
Flow cytometry analysis of Ca_15_Mab-1 against CHO/CDH15, CHO–K1, Saos-2, and C2C12. (A) CHO/CDH15 and CHO–K1 were treated with Ca_15_Mab-1 at the indicated concentrations (red) or with blocking buffer (black, negative control). (B) Saos-2 and C2C12 were treated with Ca_15_Mab-1 at the indicated concentrations (red) or with blocking buffer (black, negative control). The mAbs-treated cells were incubated with anti-mouse IgG conjugated with Alexa Fluor 488. The fluorescence data were collected using the SA3800 Cell Analyzer.

**Fig. S1:**
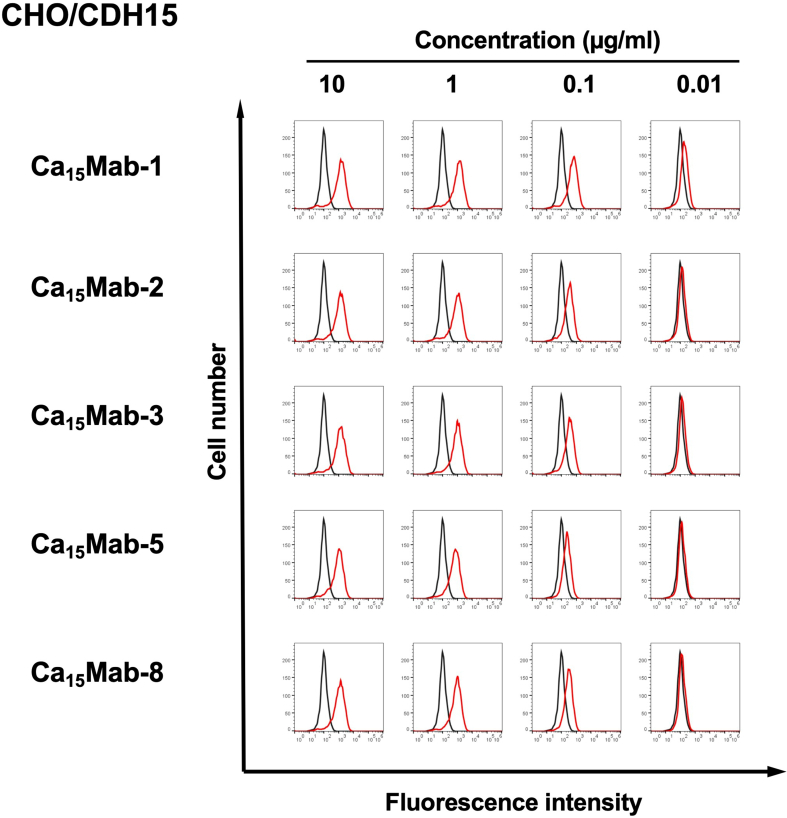
Flow cytometry analysis of Ca_15_Mabs against CHO/CDH15. CHO/CDH15 was treated with Ca_15_Mabs at the indicated concentrations (red) or with blocking buffer (black). The mAbs-treated cells were incubated with anti-mouse IgG conjugated with Alexa Fluor 488. The fluorescence data were collected using the SA3800 Cell Analyzer. The data of Ca_15_Mab-1 is represented in Fig. 2.

**Fig. S3:**
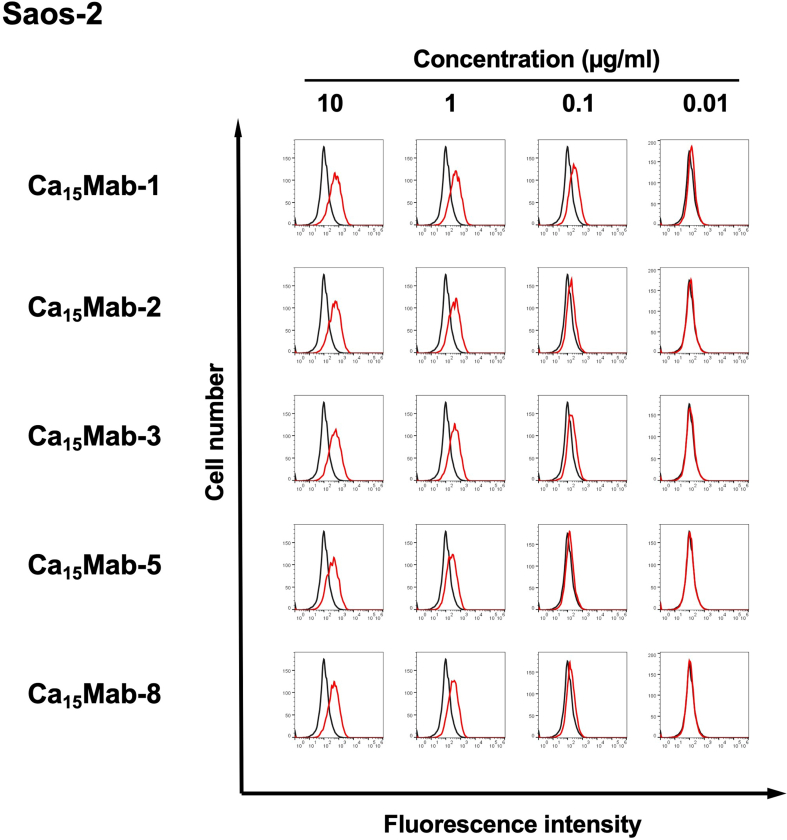
Saos-2 was treated with Ca_15_Mabs at the indicated concentrations (red) or with blocking buffer (black). The mAbs-treated cells were incubated with anti-mouse IgG conjugated with Alexa Fluor 488. The fluorescence data were collected using the SA3800 Cell Analyzer. The data of Ca_15_Mab-1 is represented in Fig. 2.

**Fig. S4:**
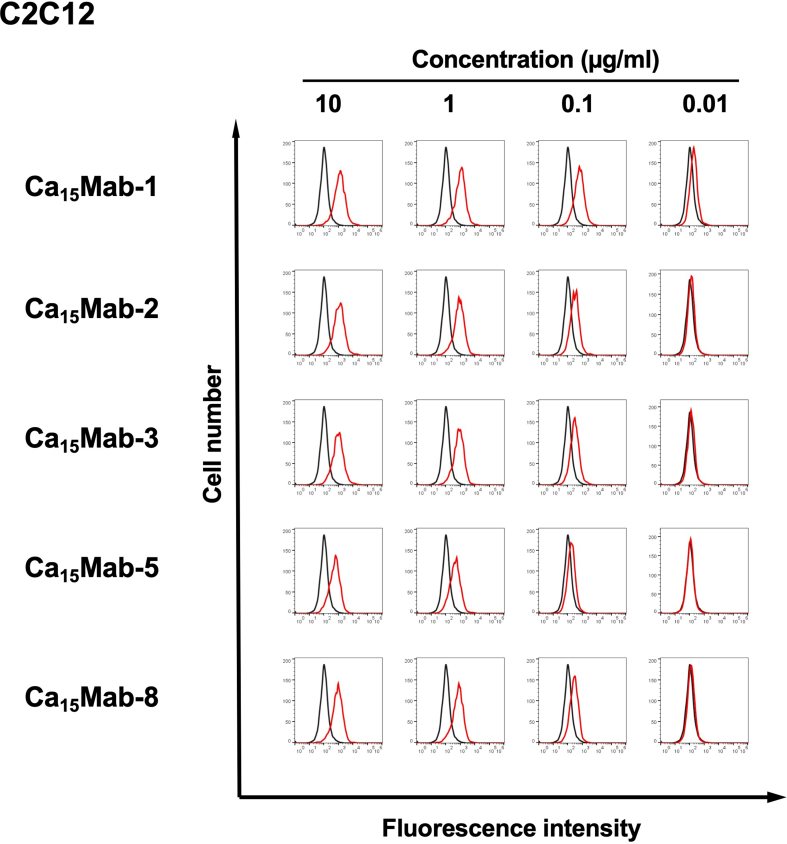
Flow cytometry analysis of Ca_15_Mabs against C2C12. C2C12 was treated with Ca_15_Mabs at the indicated concentrations (red) or with blocking buffer (black). The mAbs-treated cells were incubated with anti-mouse IgG conjugated with Alexa Fluor 488. The fluorescence data were collected using the SA3800 Cell Analyzer. The data of Ca_15_Mab-1 is represented in Fig. 2.

We conducted flow cytometry using the Ca_15_Mabs against CHO/CDH15 and CHO–K1 cells. The Ca_15_Mabs recognized CHO/CDH15 in a dose-dependent manner (Supplementary Fig. 1) from 10 to 0.01 μg/mL but did not recognize CHO–K1 even at 10 μg/mL (Supplementary Fig. 2). Since Ca_15_Mab-1 exhibits a superior reactivity among Ca_15_Mabs (supplementary Fig. 1 and Fig. 2A), we next investigated the reactivity of Ca_15_Mab-1 against endogenous CDH15-expressing cell lines, human osteosarcoma Saos-2 and mouse myoblast C2C12. Ca_15_Mab-1 showed a dose-dependent reactivity to Saos-2 and C2C12 (Fig. 2B). Other Ca_15_Mabs also reacted with Saos-2 (Supplementary Fig. 3) and C2C12 (Supplementary Fig. 4). These results suggest that Ca_15_Mab-1 recognizes human and mouse CDH15 in flow cytometry.

### 3.3 Specificity of Ca_15_Mabs to type I cadherin-expressed CHO–K1

**Fig. 3:**
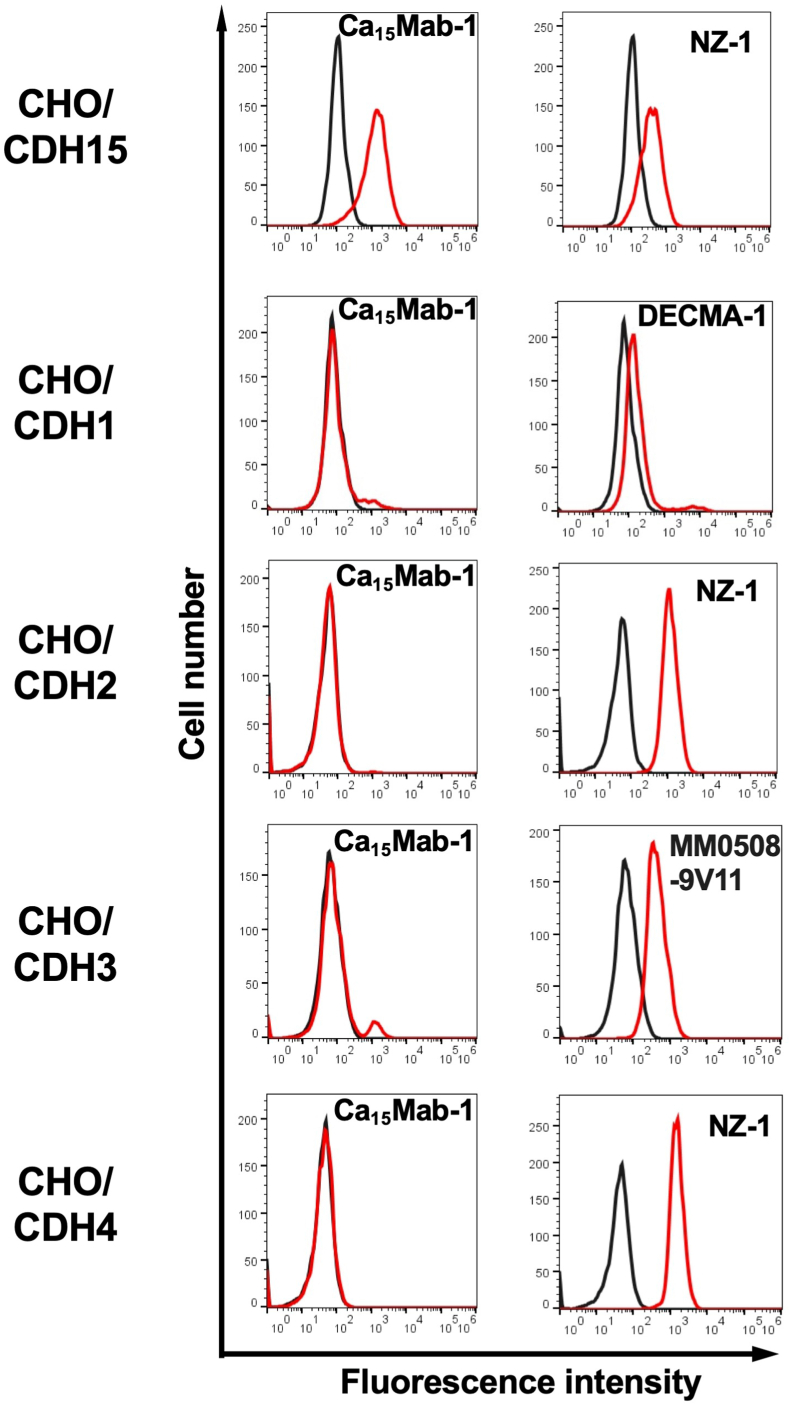
Flow cytometry analysis of Ca_15_Mab-1 in type I cadherins-expressed CHO–K1 cells. The type I cadherins (CDH1, CDH2, CDH3, CDH4, and CDH15)-expressed CHO–K1 cells were treated with 10 μg/mL of Ca_15_Mab-1 (red) or with control blocking buffer (black, negative control), followed by treatment with anti-mouse IgG conjugated with Alexa Fluor 488. Each cadherin expression was confirmed by 10 μg/mL of an anti-CDH1 mAb (clone DECMA-1), 1 μg/mL of an anti-CDH3 mAb (clone MM0508-9V11), and 1 μg/mL of an anti-PA16 tag mAb (clone NZ-1) to detect PA16-tagged CDH2, CDH4, and CDH15, followed by the treatment with anti-mouse IgG or anti-rat IgG conjugated with Alexa Fluor 488. The fluorescence data were collected using the SA3800 Cell Analyzer.

**Fig. S5:**
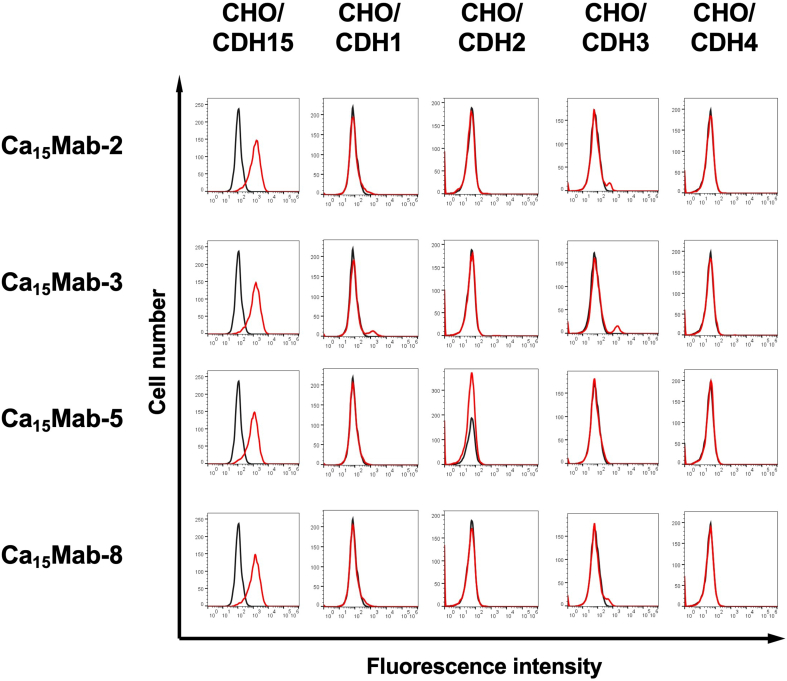
Flow cytometry analysis of Ca_15_Mabs in type I cadherins-expressed CHO–K1 cells The type I cadherins (CDH1, CDH2, CDH3, CDH4, and CDH15) expressed in CHO–K1 were treated with 10 μg/mL of Ca_15_Mab-2, Ca_15_Mab-3, Ca_15_Mab-5, Ca_15_Mab-8 (red) or control blocking buffer (black line), followed by treatment with anti-mouse IgG conjugated with Alexa Fluor 488. Each cadherin expression was confirmed in Fig. 3.

CDH15 is classified into type I cadherin, which includes CDH1/E-cadherin, CDH2/N-cadherin, CDH3/P-cadherin, CDH4/R-cadherin, and CDH15/M-cadherin [5] We established each type I cadherin-expressed CHO–K1, and the specificity of Ca_15_Mabs to type I cadherins was determined. As shown in Fig. 3, Ca_15_Mab-1 recognized CHO/CDH15, and did not react with other type I cadherins-expressed CHO–K1. Other Ca_15_Mabs also showed the same reactivity as Ca_15_Mab-1 (Supplementary Fig. 5). These results indicate that Ca_15_Mab-1 is a specific mAb against CDH15 among type I cadherins.

### 3.4 Determination of K_D_ values of Ca_15_Mab-1 by flow cytometry

**Fig. 4:**
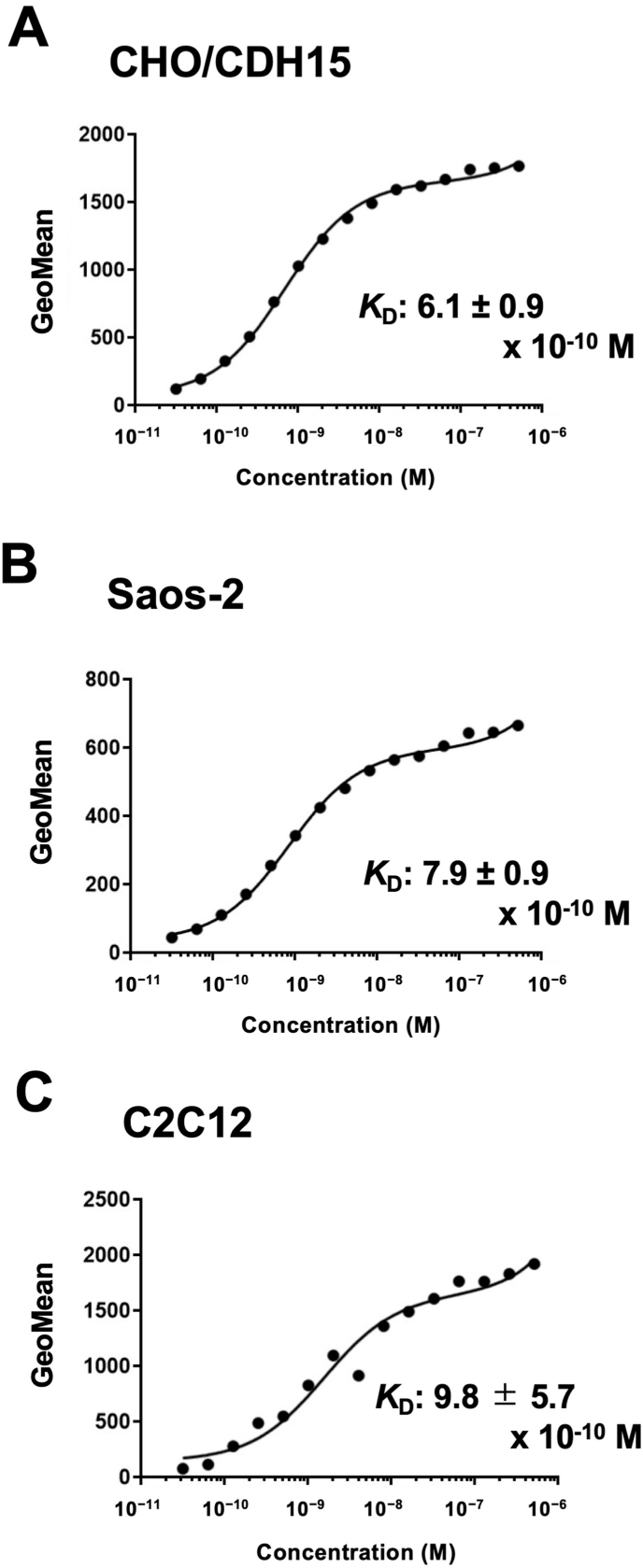
Measurement of binding affinity of Ca_15_Mab-1. CHO/CDH15 (A), Saos-2 (B), and C2C12 (C) were treated with serially diluted Ca_15_Mab-1, followed by anti-mouse IgG conjugated with Alexa Fluor 488. The fluorescence data were analyzed using the BD FACSLylic. The *K*_D_ values were determined using GraphPad PRISM 6. The average *K*_D_ values (± *standard deviation*) from three independent measurements were calculated by GraphPad PRISM 6 software. The representative graphs were shown.

**Fig. S6:**
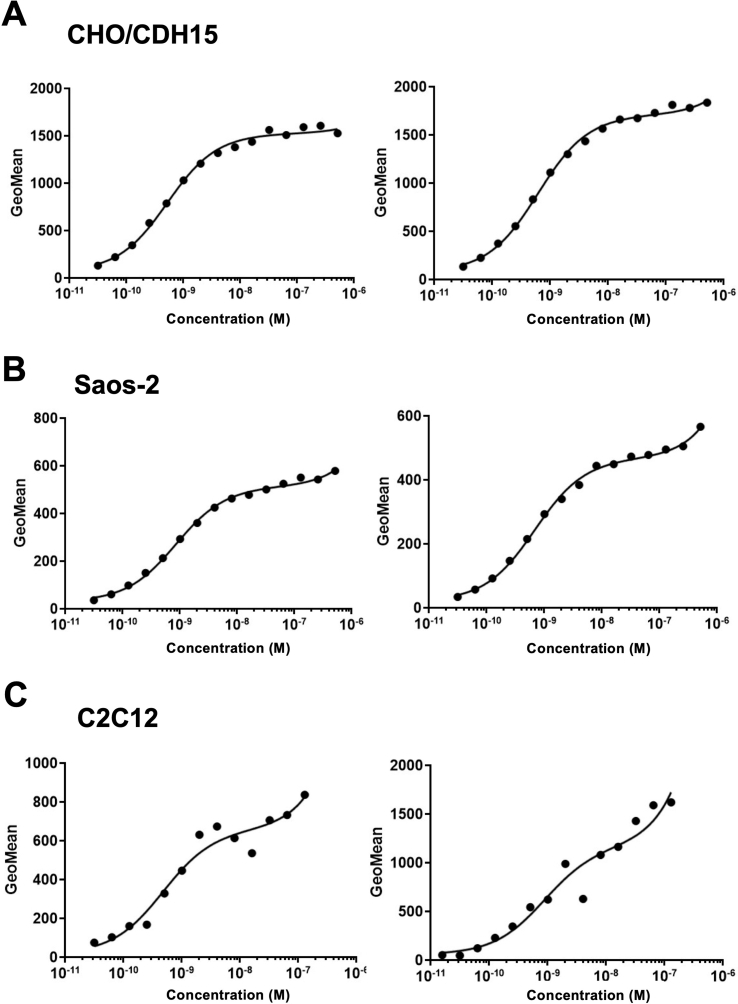
Additional two independent measurements of the binding affinity of Ca_15_Mab-1. CHO/CDH15 (A), Saos-2 (B), and C2C12 (C) were treated with mAbs serially diluted at the indicated concentrations.

We performed flow cytometry to determine the binding affinity of Ca_15_Mab-1. The fitting binding isotherms of Ca_15_Mab-1 to CHO/CDH15, Saos-2, and C2C12 were shown in Fig. 4. The replicates were shown in Supplementary Fig. 6. The *K*_D_ values of Ca_15_Mab-1 for CHO/CDH15, Saos-2, and C2C12 were (6.1 ± 0.9) × 10^−10^ M, (7.9 ± 0.9) × 10^−10^ M, and (9.8 ± 5.6) × 10^−10^ M, respectively (Fig. 4). These results showed that Ca_15_Mab-1 possesses high affinity to CDH15-positive cells.

### 3.5 Immunoblotting using Ca_15_Mab-1

**Fig. 5:**
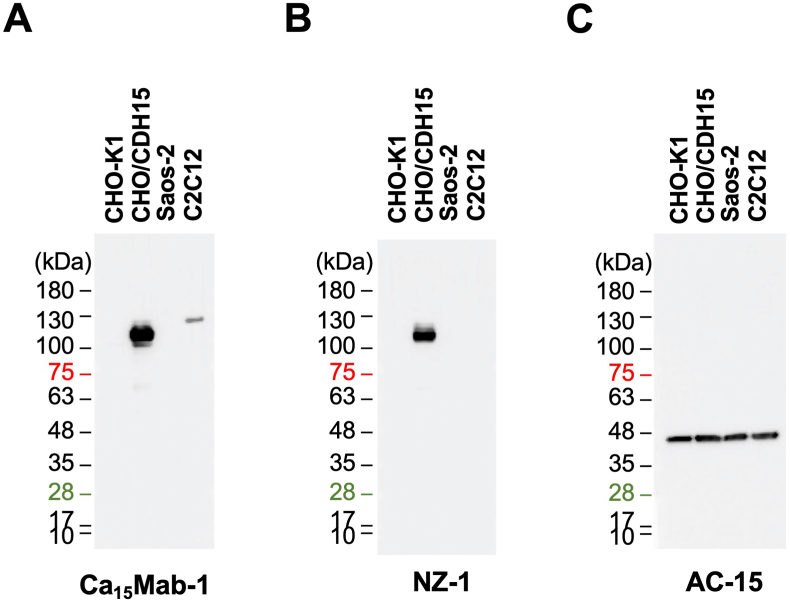
Immunoblotting using Ca_15_Mab-1. The cell lysate (10 μg/lane) of CHO–K1, CHO/CDH15, Saos-2, and C2C12 were electrophoresed and transferred onto polyvinylidene difluoride membranes. The membranes were incubated with 1 μg/mL of Ca_15_Mab-1 (A), 1 μg/mL of NZ-1 (B), and 1 μg/mL of AC-15 (an anti-β-actin mAb) (C), followed by the treatment with anti-mouse or anti-rat IgG conjugated with horseradish peroxidase.

**Fig. S7:**
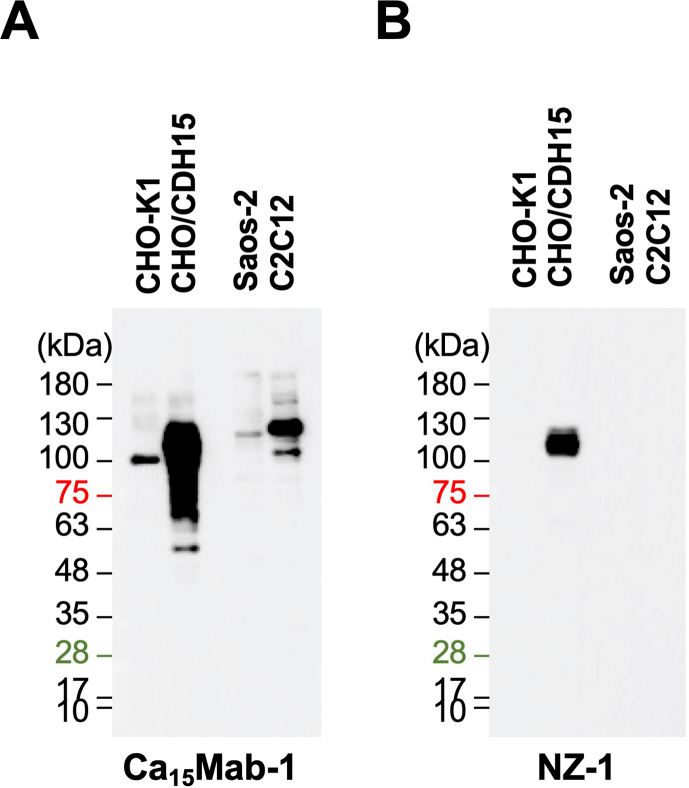
Immunoblotting using a high concentration of Ca_15_Mab-1 The cell lysate (10 μg/lane) of CHO–K1, CHO/CDH15, Saos-2, and C2C12 were electrophoresed and transferred onto polyvinylidene difluoride membranes. The membranes were incubated with 10 μg/mL of Ca_15_Mab-1 (A) and 1 μg/mL of NZ-1 (B), followed by treatment with anti-mouse or anti-rat IgG conjugated with horseradish peroxidase.

We next examined whether Ca_15_Mab-1 is suitable for immunoblotting. Whole-cell lysates of CHO–K1, CHO/CDH15, Saos-2, and C2C12 were utilized. Ca_15_Mab-1 (1 μg/mL) exhibited a clear band around 110 kDa in CHO/CDH15 but not in CHO–K1 (Fig. 5A). An anti-PA16-tag mAb (NZ-1) also detected a similar band (Fig. 5B). Furthermore, Ca_15_Mab-1 was able to detect endogenous CDH15 in C2C12 at a slightly higher molecular weight compared to that of CHO/CDH15 (Fig. 5A). We could also detect a band in Saos-2 using a higher concentration of Ca_15_Mab-1 (10 μg/mL, Supplementary Fig. 7). An anti-β-actin mAb (AC-15) served as an internal control (Fig. 5C). These results indicate that Ca_15_Mab-1 can detect exogenous and endogenous CDH15 in immunoblotting.

### 3.6 IHC using Ca_15_Mab-1 in formalin-fixed paraffin-embedded cell blocks

**Fig. 6:**
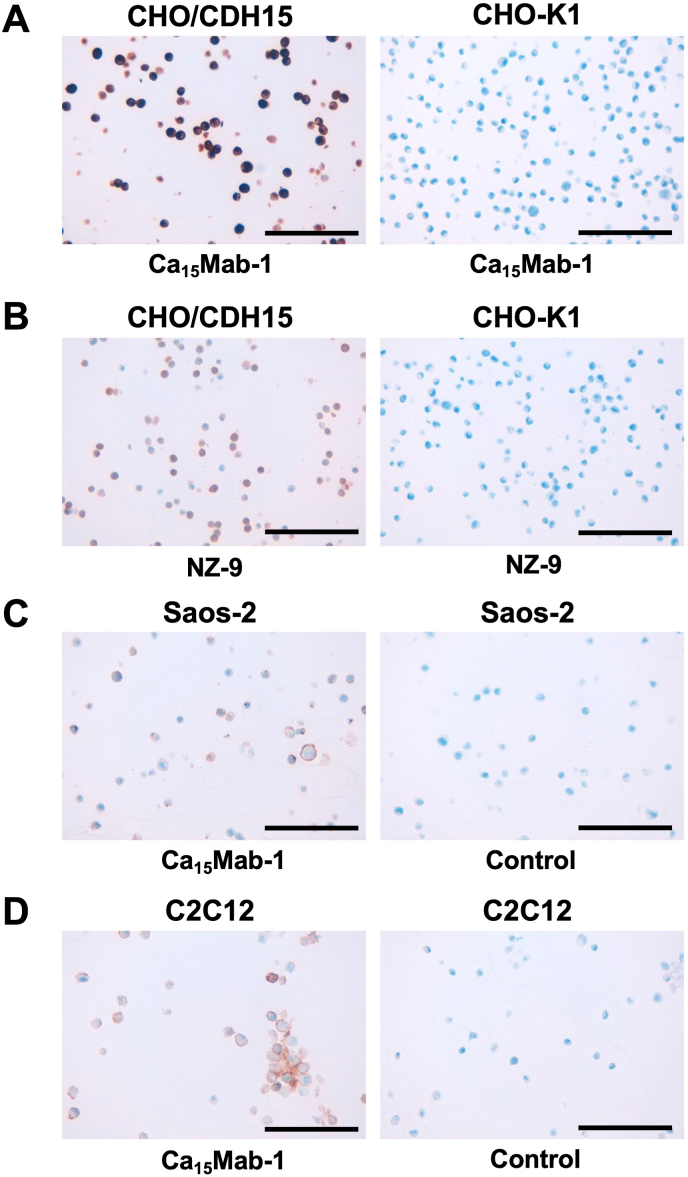
Immunohistochemistry using Ca_15_Mab-1 in formalin-fixed paraffin-embedded cell blocks. (A) CHO/CDH15 and CHO–K1 sections were treated with 0.1 μg/mL of Ca_15_Mab-1. (B) CHO/CDH15 and CHO–K1 sections were treated with 1 μg/mL of NZ-9. (C) The sections of Saos-2 were treated with 1 μg/mL of Ca_15_Mab-1 or control (without primary Ab). (D) The sections of C2C12 were treated with 1 μg/mL of Ca_15_Mab-1 or control (without primary Ab). The staining was performed using BenchMark ULTRA PLUS with the ultraView Universal DAB Detection Kit. Scale bar = 100 μm.

We investigated whether Ca_15_Mab-1 suits the IHC of FFPE sections of CHO–K1, CHO/CDH15, Saos-2, and C2C12. Both intense membranous and cytoplasmic staining by Ca_15_Mab-1 were detected in CHO/CDH15 but not in CHO–K1 (Fig. 6A). A rat-mouse anti-PA16-tag mAb, NZ-9 also reacted with CHO/CDH15, but not CHO–K1 (Fig. 6B). Furthermore, membranous staining by Ca_15_Mab-1 was observed in Saos-2 (Fig. 6C) and C2C12 (Fig. 6D). These results indicate that Ca_15_Mab-1 can detect exogenous and endogenous CDH15 in FFPE cell samples.

## 4 Discussion

The isolation of purified stem cell populations has dramatically changed the field of stem cell biology and has become a key approach in various tissues and organisms. Skeletal muscle SCs are the most extensively studied stem cells in mammalian systems, and their targeted isolation has enabled cellular and molecular analyses. Quiescent muscle SCs are initially defined by their anatomical position to the myofiber [36]. They can be identified by molecular markers, such as PAX7, CDH15, syndecan 3, syndecan 4, α7β1 integrin, VCAM-1, caveolin 1, and CXC chemokine receptor 4 (CXCR4) [18,37].

Studies have reported the procedures for isolating SCs from the limb muscles using fluorescence-activated cell sorting. Several mAbs against VCAM-1 [38], α7 integrin [39], and β1 integrin/CXCR4 [40] have been used for the isolation of SCs. Since these cell surface markers are expressed in other cell types, such as vascular endothelial cells and hematopoietic cells, the negative selection using anti-CD31 and/or anti-CD45 mAbs is also essential [37]. However, these protocols still have the possibility of isolating vascular endothelial cells, mesenchymal stem cells, and hematopoietic cells from muscle tissue [37]. Given that the expression of CDH15 is limited in SCs compared to other markers [12,13], the isolation of CDH15-positive cells is considered essential for SC research. However, the isolation of CDH15-positive SCs has not been conducted, likely due to the absence of an anti-CDH15 mAb for flow cytometry.

This study first reported the anti-CDH15 mAbs for flow cytometry using the CBIS method (Fig. 1). An anti-CDH15 mAb, Ca_15_Mab-1, recognized both exogenous and endogenous CDH15 in flow cytometry with high affinity (Fig. 2, Fig. 3, Fig. 4). Furthermore, Ca_15_Mab-1 is suitable for immunoblotting (Fig. 5) and IHC (Fig. 6). Therefore, Ca_15_Mab-1 is highly versatile for basic research and would contribute to SC biology in muscles.

Ca_15_Mab-1 also recognized mouse myoblast C2C12 in flow cytometry (Fig. 2, Fig. 4), immunoblotting (Fig. 5), and IHC (Fig. 6). *Cdh15* was first isolated from C2C12 cDNA [10]. C2C12 has been used in skeletal muscle research as a cell culture model [41]. C2C12 cells show potent proliferation and differentiation abilities. In low serum conditions, the cells undergo differentiation into myotubes and myofibers with cell fusion and aggregation of nuclei [42]. C2C12 has been used in various studies, such as myotube atrophy in a cancer cachexia model [43,44]. Therefore, Ca_15_Mab-1 will contribute to the research on myotube and myofiber differentiation in the future.

During aging, muscle regenerative ability is hampered due to impaired SC function [45]. A notable reduction in the number of PAX7-positive SCs has been reported in aged skeletal muscle under homeostatic conditions [46,47]. An age-dependent change in the SC niche results in the loss of ability to retain the quiescent state [23]. In several mouse models, the effects of the genetic depletion of SCs have been reported. Depletion of SCs using the *Pax7*^*CreER/+*^; *Rosa26*^*DTA/+*^ mice (DTA: diphtheria toxin A) in young adult mice impairs regeneration throughout the rest of their lives. However, the size of SC-depleted muscles was standard despite low regenerative capacity, but the muscle had increased fibrosis [48]. These results suggest that lifelong depletion of SCs did not accelerate nor exacerbate sarcopenia, the age-associated loss of skeletal muscle mass and strength. SCs were not essential to maintaining muscle size or fiber type composition during aging in mice.

**Fig. S8:**
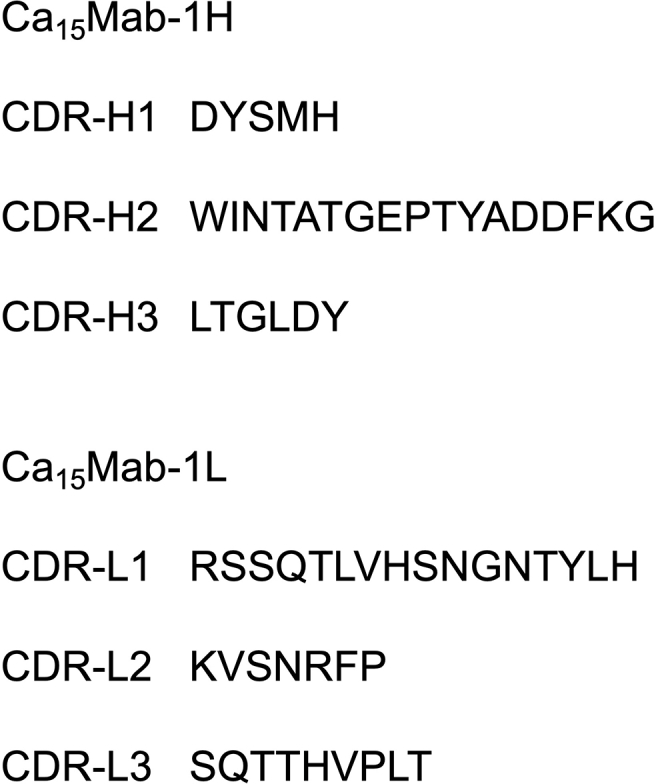
Amino acid sequence of complementarity-determining regions of Ca_15_Mab-1. The cDNAs of Ca_15_Mab-1 variable regions were cloned, and the amino acid sequences of complementarity-determining regions (CDRs) were determined.

A study reported that depletion of SCs suppressed the progression of muscular dystrophy. The Sgcd^−/−^ mouse is a model of limb-girdle muscular dystrophy type 2F, devastating and life-limiting muscular dystrophy with no cure [49]. Genetic depletion of SCs in Sgcd^−/−^ mice reduced the features of muscular dystrophy, including improved histopathology, increased sarcolemmal stability, and augmented muscle performance [50]. Mechanistically, SC-mediated induction of the fetal genes leads to sarcolemmal instability in myofibers during muscular dystrophy [50]. Since the depletion of SCs was achieved through a complicated genetic background (Sgcd^−/−^; Mapk3^−/−^; Mapk1^f/f−Pax7Cre−ER^), a simple depletion of SCs using mAbs is thought to be essential to obtain a proof of concept for the therapy of muscular dystrophy. Since CDH15 expression is localized to SCs [12,13], CDH15 is thought to be an essential target. We have cloned the cDNA of Ca_15_Mab-1 variable regions and showed amino acid sequences of complementarity-determining regions (Supplementary Fig. 8). We previously changed the isotype of mAbs into mouse IgG_2a_ or human IgG_1_ to obtain antibody-dependent cellular cytotoxicity (ADCC), which is mainly used for the evaluation of antitumor activities in mouse xenograft models [51,52]. Since the subclass of Ca_15_Mab-1 is mouse IgG_1_, it does not exert ADCC. A class-switched Ca_15_Mab-1 will be useful in investigating the effect of depletion of SCs in the mouse model of muscular dystrophy.

The expression of CDH15 in cancers has not been explored. Ca_15_Mab-1 recognized osteosarcoma Saos-2 in flow cytometry (Fig. 2, Fig. 4), immunoblotting (Supplementary Fig. 7), and IHC (Fig. 6). Since we have not yet investigated other types of sarcomas, such as rhabdomyosarcoma, it is worthwhile to identify more tumors expressing CDH15. Ca_15_Mab-1 and its derivatives could contribute to cancer cell biology and cancer therapy in the future.

## CRediT authorship contribution statement

**Rena Ubukata:** Investigation. **Hiroyuki Suzuki:** Writing – original draft, Funding acquisition. **Tomohiro Tanaka:** Investigation, Funding acquisition. **Mika K. Kaneko:** Conceptualization. **Yukinari Kato:** Project administration, Conceptualization, Writing – review & editing, Funding acquisition.

## Author disclosure statement

The authors have no conflict of interest.

## Funding information

This research was supported in part by 10.13039/100009619Japan Agency for Medical Research and Development (AMED) under Grant Numbers: JP25am0521010 (to Y.K.), JP25ama121008 (to Y.K.), JP25ama221339 (to Y.K.), and JP25bm1123027 (to Y.K.), and by the 10.13039/501100001691Japan Society for the Promotion of Science (JSPS) Grants-in-Aid for Scientific Research (KAKENHI) grant nos. 22K06995 (to H.S), 24K18268 (to T.T.), and 25K10553 (to Y.K.).

## Declaration of competing interest

The authors declare that they have no known competing financial interests or personal relationships that could have appeared to influence the work reported in this paper.

## Figures and Tables

**Fig. S:**
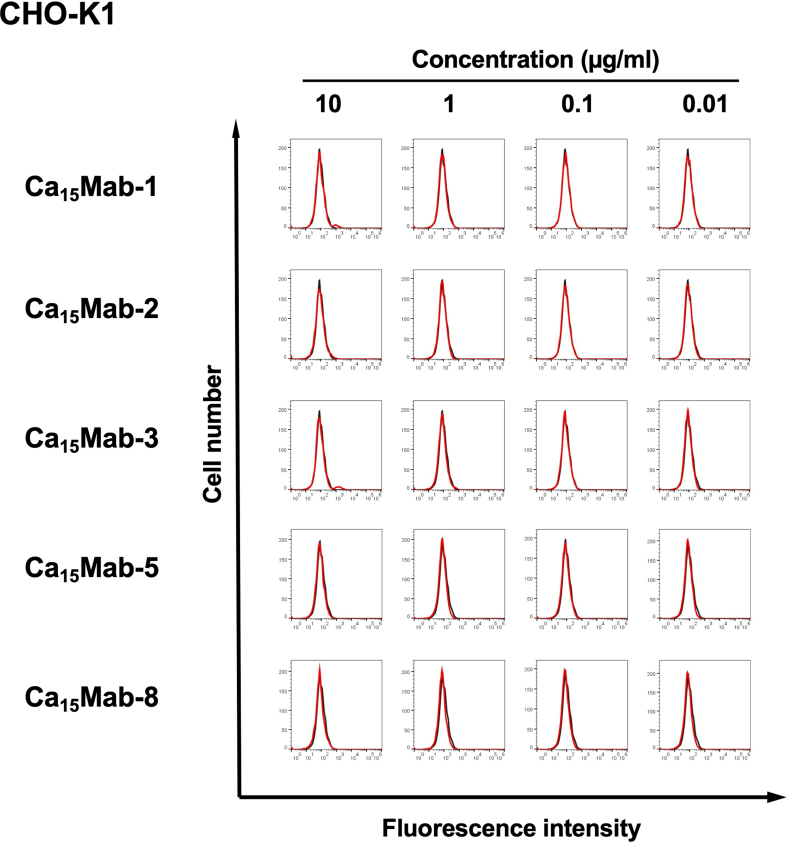
2Flow cytometry analysis of Ca_15_Mabs against CHO–K1. CHO–K1 was treated with Ca_15_Mabs at the indicated concentrations (red) or with blocking buffer (black). The mAbs-treated cells were incubated with anti-mouse IgG conjugated with Alexa Fluor 488. The fluorescence data were collected using the SA3800 Cell Analyzer. The data of Ca_15_Mab-1 is represented in Fig. 2.
